# C-COMPASS: protocol for a quasi-experimental hybrid type I effectiveness-implementation study of community-based compassionate care after stillbirth in India

**DOI:** 10.3389/frhs.2026.1879371

**Published:** 2026-07-14

**Authors:** Barsha Gadapani Pathak, Sonia Maurya, Pranay Vats, Sanjay Sharma, Bhupendra Singh, Jyothi Singhal, Sarmila Mazumder

**Affiliations:** 1 Society for Applied Studies, New Delhi, Delhi, India; 2 Centre for Intervention Science in Maternal and Child Health, Centre for International Health, Department of Global Public Health and Primary Care, University of Bergen, Bergen, Norway; 3 District Health Authority, Palwal, Haryana, India

**Keywords:** community health workers, difference-in-differences, early neonatal death, Edinburgh postnatal depression scale, implementation science, incremental cost, India, perinatal loss

## Abstract

**Background:**

India contributes approximately one-quarter of the global stillbirth burden, with an estimated 460,000 stillbirths annually-one of the highest absolute burdens globally. Despite this, women experiencing stillbirth or early neonatal death face a high and largely unaddressed burden of postpartum depression, complicated grief, and functional impairment in the weeks and months following loss. No structured, community-level psychosocial support protocol is currently integrated into India's public health system, leaving Accredited Social Health Activists (ASHAs)-who routinely visit women after birth- without standardised guidance for compassionate care after perinatal loss.

**Methods:**

This protocol describes a quasi-experimental hybrid type I effectiveness-implementation study embedded within an ongoing implementation research project, SHRiSTI, aiming to reduce stillbirths in India. The Community Continuum of Care for Compassionate Support After Stillbirth (C-COMPASS) is a co-designed, community health worker–delivered counselling intervention for women experiencing stillbirth and early neonatal death. The intervention comprises five structured contacts: a telephonic follow-up within 72 h of discharge, and on Day 7, in-person visits on Day 14 and Day 21, and a Day 42 postpartum review, supported by a red-flag referral pathway. The study will use a quasi-experimental Difference-in-Differences (DiD) analytical design across four administrative blocks (two intervention, two control), with a pre-intervention observation period (approximately 10–12 weeks) and a post-intervention period (approximately 26 weeks). This design was chosen in place of a cluster-randomised controlled trial because block-level allocation is operationally determined within the existing SHRiSTI programme. The primary effectiveness outcome will be mean Edinburgh Postnatal Depression Scale (EPDS) score at 60 days postpartum, measured by independent assessors and analysed using a linear DiD model with block-level fixed effects. An expected sample of approximately 56 women per arm in the post-intervention period provides 81% power to detect a clinically meaningful difference of 3.5 EPDS points with a total anticipated enrolment of approximately 146 women. Secondary outcomes will include binary EPDS score thresholds, grief symptoms, functional disability, and incremental cost per woman counselled. Early neonatal deaths within 24 h will be included in a pre-specified sensitivity analysis. Implementation outcomes will be assessed using the Proctor et al. taxonomy.

**Discussion:**

C-COMPASS is among the first community-based interventions in India designed to address psychosocial care following stillbirth through frontline health workers. If demonstrated to be effective and feasible, this model may provide a scalable, low-cost approach for integration into India's ASHA-based maternal health system.

**Clinical Trial Registration:** The primary implementation research was registered prospectively in the Clinical Trial Registry of India (CTRI): CTRI/2024/07/069796 [Registered on: 02/07/2024].

## 1 Introduction

Stillbirth, defined by the World Health Organization as foetal death at 28 or more completed weeks of gestation, remains one of the most prevalent yet under-addressed outcomes in global maternal and newborn health. An estimated 1.9 million stillbirths occur annually, with over three-quarters concentrated in sub-Saharan Africa and South Asia (1). India alone contributes approximately 460,000 stillbirths each year (2, 3), accounting for nearly one-quarter of the global burden, despite substantial progress in reducing maternal and neonatal mortality over the past decade (2, 3).

**Table 1:** C-COMPASS contact schedule.

Contact	Timing	Mode	Core components
Contact 1: Early follow-up	Within 72 h of discharge	Telephone call (≥10 min)	Emotional check-in; follow-up on lactation/breast health; direct enquiry for self-harm (using scripted probe); red-flag screening
Contact 2: Check-in	Day 7 (±1 day)	Telephone call	Emotional acknowledgement and active listening; guidance on lactation suppression; assess bleeding, fever and breast pain; family engagement; provide Tele-MANAS helpline information (1800-891-4416); confirm Day 14 visit
Visit 1: Recovery	Day 14 (±2 days)	In-person home visit	First in-person visit after two telephonic counselling sessions; assess physical recovery and grief; breast health check; **Circle 1-** Heart/Feelings; **Circle 2:** Mind/Positive Thinking; family counselling; initiation of contraceptive counselling; identify and refer red flags
Visit 2: Continued recovery	Day 21 (±2 days)	In-person home visit	Assess recovery; check for physical and emotional signs of grief and breast engorgement; reinforce family support; counsel on pregnancy spacing; identify women needing further support from district psychiatrist
Contact 3: Postpartum review	Day 42 (±2 days)	Telephone call	Postpartum evaluation; follow-up on emotional wellbeing; precise contraceptive counselling; preconception planning; update ASHA registers; **Circle 3-Support** and update ASHA registers.

Beyond mortality, stillbirth is associated with profound and enduring psychosocial consequences. Women who experience stillbirth face a markedly increased risk of postpartum depressive disorder, with prevalence estimates ranging from 25% to 50% in low- and middle-income countries (4), substantially higher than among women with live births (4, 5). In addition to depression, women frequently experience complicated grief, persistent emotional distress, and functional impairment affecting social, familial, and occupational domains, with effects often extending beyond one year (6, 7). These consequences are exacerbated in contexts where perinatal loss is accompanied by stigma, blame directed at the mother, and limited access to mental health services, conditions widely documented in rural North India (8, 9).

Importantly, the psychosocial burden of stillbirth exists within a broader continuum of perinatal loss that includes early neonatal death, late miscarriage, and preterm death. Evidence suggests that grief intensity and depressive symptoms following stillbirth and early neonatal death are clinically comparable during the early postpartum period (4, 5, 7). In India, both outcomes are captured under the National Health Mission (NHM) perinatal mortality framework and together affect an estimated 700,000 families annually. Despite this substantial burden, structured psychosocial support for women following perinatal loss is not systematically integrated into India's public health system. Existing postnatal care frameworks primarily focus on women with live births, and frontline community health workers, Accredited Social Health Activists (ASHAs), receive no standardized guidance for providing care after stillbirth (10). As a result, support following perinatal loss remains informal, inconsistent, and largely undocumented. Women are often discharged within 24–48 h of delivery with minimal counselling and return to home environments where grief may be compounded by social silence, pressure, or blame. Evidence from India further highlights a critical gap in structured grief care and bereavement support at the community level, despite the established role of community health workers in delivering psychosocial and behaviour-change interventions (11–13). While several international bereavement care protocols exist, including those developed by the International Stillbirth Alliance, the American College of Obstetricians and Gynaecologists, and SANDS, these are designed for high-resource facility settings and are not readily adaptable to community-based delivery in low-resource contexts.

To address this gap, the Community Continuum of Care for Compassionate Support After Stillbirth (C-COMPASS) was co-designed by research team and other stakeholders within the SHRiSTI (Strategies to Help in Optimal Pregnancy Outcomes and Reduce Stillbirths in India) implementation research project. This care protocol aims to operationalize compassionate care into a structured, scalable, community-based response delivered through ASHAs as the primary frontline implementers. Supervisory and first-level clinical support will be provided at Health and Wellness Centres (HWCs), the most accessible level of healthcare in India, with specialist physicians available for management of complex physical and psychological complications. This study describes the protocol for evaluating C-COMPASS using an quasi-experimental effectiveness-implementation design to assess both its impact on maternal mental health outcomes and its feasibility within the existing health system.

## 2 Methodology

### 2.1 Study design and site

This is a prospective, quasi-experimental, Hybrid Type I effectiveness-implementation study using a Difference-in-Differences (DiD) analytical design (14). The Hybrid Type I classification-where effectiveness is the primary scientific aim and implementation outcomes are assessed concurrently-is appropriate, given that C-COMPASS has not previously been evaluated. Implementation outcomes will be assessed using the Proctor et al. (13) taxonomy rather than Reach effectiveness- adoption implementation maintenance (RE-AIM), as the latter presupposes prior effectiveness evidence (13). C-COMPASS is embedded within the SHRiSTI (Strategies to Help in Optimal Pregnancy Outcomes and Reduce Stillbirths in India) implementation research programme, which has been conducted across seven states in India since 2024. However, the C-COMPASS intervention will be implemented and evaluated only in Palwal district, Haryana, India. A cluster-randomised controlled trial is not feasible in this context because C-COMPASS is embedded within an existing programme where block-level allocation is operationally determined, ethical constraints preclude withholding an untested intervention by randomisation before evidence of effect exists, and the short enrolment window required by the SHRiSTI project's timeline cannot accommodate the lead time needed for stratified block randomisation; the DiD design provides a rigorous quasi-experimental alternative by controlling for time-invariant block-level differences through fixed effects and leveraging the natural experiment created by phased programme rollout (14).

Palwal is a predominantly rural district located approximately 80 kilometres south of New Delhi, with a total population of 1,289,685 (HMIS data). The district comprises six health blocks. The district has a sex ratio of 943 females per 1,000 males and a sex ratio at birth of 884 per 1,000 males. The female literacy rate is 68.3% among women aged 15–49 years, with 39.4% having completed 10 or more years of schooling (NFHS-5). The pregnancy rate is 116 per 1,000 women of reproductive age. The HMIS-reported stillbirth rate is 9.4 per 1,000 live births (266 stillbirths among 28,126 live births); however, this substantially underestimates the true burden. SHRiSTI's ASHA-based community surveillance documents a stillbirth rate of 17.2 per 1,000 total births. The perinatal mortality rate reported in HMIS is 14.9 per 1,000 live births which as per the SHRiSTI's ASHA-based community surveillance is around 29.2 per 1,000 live births.

The district health system comprises 1,019 ASHA workers, one District Hospital, one Sub-District Hospital, six Community Health Centres, 14 Primary Health Centres, three Urban PHCs, and 96 Health and Wellness Centres/Sub-centres (92 rural and four urban). Within SHRiSTI, a community-based stillbirth surveillance system has been established in collaboration with district health authorities, with all ASHAs actively reporting across all blocks of Palwal district.

### 2.2 Study hypothesis

C-COMPASS, delivered by ASHAs to women following stillbirth, will result in significantly lower postpartum depressive symptoms at 60 days postpartum compared to routine postnatal care, and will demonstrate acceptable feasibility and implementation performance within the existing district health system**.**

### 2.3 Study Aim

To evaluate the effectiveness and implementation performance of C-COMPASS, a community-based structured compassionate care protocol, among women experiencing stillbirth in Palwal district, Haryana.

#### 2.3.1 Objectives

##### 2.3.1.1 Primary

To assess the effect of C-COMPASS on postpartum depressive symptoms, measured by mean Edinburgh Postnatal Depression Scale (EPDS) total score at Day 60 postpartum, compared to routine care.

##### 2.3.1.2 Secondary

- To assess the effect on functional impairment among women with stillbirths at Day 60 postpartum.
- To assess the effect on grief distress among women with stillbirths at Day 60 postpartum.
- To estimate the proportion of women screening positive for probable depression (EPDS ≥13) in each arm, as exploratory evidence for future trial design
- To explore whether effects remain consistent when early neonatal deaths (<24 h) are included alongside stillbirth.
- To assess the feasibility of C-COMPASS delivery within the existing ASHA system, measured by 72 h ASHA visit coverage
- To estimate the incremental cost of C-COMPASS per woman counselled compared to routine care.

### 2.4 Study period

The study comprises a pre-intervention observation period (March–May 2026, approximately 10–12 weeks) and a post-intervention period (May–November 2026, approximately 26 weeks), encompassing the July–October seasonal peak in stillbirths (5.2–5.6 per block per month). Data collection will be completed by December 2026.

### 2.5 Study population and eligibility

*Inclusion criteria:* All women residing in the four study blocks who experience a stillbirth (intrauterine foetal death at ≥28 completed weeks of gestation) during the study period, are able to provide informed consent, and are reachable for an ASHA home visit.

*Exclusion criteria:* Women with severe cognitive impairment precluding informed consent, or those who explicitly decline participation.

#### 2.5.1 Sensitivity analysis population

A pre-specified sensitivity analysis will additionally include women experiencing early neonatal death within 24 h of live birth in the study blocks during the same period.

The physiology of postpartum recovery is identical to that following stillbirth, and existing evidence demonstrates overlapping grief and depressive symptom profiles during the first six postpartum weeks (25, 26). However, as C-COMPASS was co-designed around the stillbirth experience-where the mother has not held a living infant-the counselling narrative requires sensitivity adaptation for women who briefly held a live-born baby.

Accordingly, early neonatal deaths will be enrolled separately with a subgroup identifier, excluded from the primary analysis, and included only in the pre-specified sensitivity analysis.

#### 2.5.2 Target population

The intervention delivery comprises frontline community health workers, including Accredited Social Health Activists (ASHAs), Auxiliary Nurse Midwives (ANMs), and Community Health Officers (CHOs).

ASHAs serve as the primary implementers of the C-COMPASS intervention at the community level, delivering structured counselling during postnatal home visits. ANMs and CHOs provide supervisory and referral support, including initial clinical assessment and coordination of care. Where indicated, women identified with clinical or psychological complications are referred to higher-level providers, including obstetricians/gynaecologists and district-level mental health specialists (e.g., psychiatrists), as part of the established referral pathway.

### 2.6 The C-COMPASS protocol

C-COMPASS is a structured, phased, community-based continuum of care co-designed through iterative consultation cycles within the SHRiSTI project. The co-design process involved district psychiatrists, programme managers and other district health authorities, ASHA workers, ANMs, CHOs, the district ASHA coordinator, specialist like obstetricians, implementation science experts, and national stakeholders engaged in stillbirth surveillance and guidelines. Additionally, insights were also obtained from the bereaved women through social autopsy.

The intervention is grounded in three core principles:

1. grief after stillbirth is a normal human response that requires acknowledgement rather than medicalisation;
2. structured support must begin early, given the acute psychosocial vulnerability in the first 72 h after returning home; and
3. the ASHA-community relationship, built on geographic proximity and trust, provides the most appropriate platform for delivering community-based support.

In addition, the intervention adopts an integrated approach that combines emotional support with basic physical health assessment and a structured referral pathway for identified red-flag conditions, ensuring continuity of care across community and facility levels.

The intervention architecture is designed to be adaptable to early neonatal death without requiring fundamental redesign.

#### 2.6.1 Intervention deliverers

The intervention will be delivered by Accredited Social Health Activists (ASHAs), with support from the study team supervisors. ASHAs were selected as the primary implementers as they are trained under the National Health Mission (NHM) and function as the first point of contact between communities and the formal health system.

ASHAs are already mandated to conduct postnatal home visits under the Home-Based Newborn Care (HBNC) programme. C-COMPASS does not require the introduction of a new cadre of workers or additional visits beyond the existing postnatal care schedule. Instead, it integrates a structured counselling protocol, targeted training, and a defined referral pathway into routine ASHA-led visits. This design enhances feasibility and scalability by leveraging existing health system infrastructure and enables assessment of whether structured psychosocial support following perinatal loss can be delivered within the current system at marginal additional cost.

#### 2.6.2 C-COMPASS comprises four structured contacts across the 42-day postpartum period

Table 2.

**Table 2:** Incremental cost analysis- components, data sources, and collection methods.

Cost component	Unit	Data source	Method of estimation
ASHA time: Contact 1 (≤72 h)	Minutes per case	ASHA self-report (end of study)	Time × imputed hourly rate
ASHA time: Day 7 call	Minutes per case	ASHA self-report	As above
ASHA time: Visit 2 (Day 14)	Minutes per case	ASHA self-report	As above
ASHA time: Visit 3 (Day 21)	Minutes per case	ASHA self-report	As above
ASHA time: Contact at 42 days	Minutes per case	ASHA self-report	As above
ASHA transport cost	INR per case	ASHA self-report	Direct expenditure
Printed materials/IEC	INR per case	Programme records	Procurement records
ASHA training (amortised)	INR per case	District records	Total training cost ÷ 3 yrs ÷ annual cases
CHO supervision time	INR per case	CHO self-report	Time×CHO salary rate
Routine care cost (control arm)	INR per case	Control block CHO records	Existing per-woman postnatal expenditure

**Table 3:** Detailed framework selection processes.

SELECT-IT purpose	TMF selected	TMF not selected	Key attribute rationale	Practical consideration
Guiding identification of determinants (barriers/facilitators)	TDF (Theoretical Domains Framework)	CFIR (Consolidated Framework for Implementation Research)	TDF focuses on individual-level behaviour change, aligning with ASHA delivery processes; however, it does not fully capture system-level barriers. Both frameworks are evidence-based.	The study team has qualitative and implementation science expertise and can be using this TDF.
Guiding design and adaptation of strategies	ERIC Taxonomy (Expert Recommendations for Implementing Change)	Implementation Mapping	ERIC provides 73 clearly defined, operationalised strategies with standardised labels and action verbs, enabling direct application in ASHA training and CHO supervision. It has strong alignment with behaviour change evidence. Implementation Mapping was considered more resource intensive.	ERIC tools are readily available and usable without extensive training. Strategies can be directly linked to determinants identified during co-design.
Guiding evaluation and causal explanation	Proctor et al. (13) taxonomy	RE-AIM framework	Proctor's taxonomy includes eight implementation outcomes (acceptability to sustainability) suitable for early-stage evaluations where effectiveness is not yet established. RE-AIM assumes prior evidence on effectiveness and dissemination readiness.	Proctor measures are well-established and align with existing data collection tools, including ASHA feasibility and evaluation matrices.
Guiding the implementation process (study design)	Curran et al. (21) Hybrid Type I	Hybrid Type II or III	Hybrid Type I prioritises effectiveness while concurrently assessing implementation, appropriate for first-time evaluation. Hybrid II/III designs assume greater emphasis on implementation, which is not suitable at this stage.	The design aligns with the Difference-in-Differences analytical approach and is widely recognised as appropriate for early implementation studies.

#### 2.6.3 Identification, notification, and referral

Facility stillbirths will be recorded by nurses in labour room registers, case sheets, and MCP cards, and the ASHA will be notified within 24 h via SMS. For home deliveries, the ASHA will directly register the event. Following notification, the ASHA will initiate the first contact. If a home visit is not feasible, the ANM or facility nurse will assume responsibility for the initial contact.

At each contact, ASHAs will assess for predefined physical and emotional red flags using operational definitions provided in the ASHA Job Aid and covered during training. Physical red flags include heavy vaginal bleeding (soaking ≥ 2 pads per hour for two consecutive hours or clots larger than a 50-paise coin); fever (≥38 °C or feeling hot to touch with rigors); wound complications (pus, suture dehiscence, or foul-smelling discharge); breast complications with fever suggesting mastitis; and seizures. Emotional red flags include: suicidal ideation, assessed at every contact using a scripted Hindi-language probe and defined as any affirmative response or spontaneous expression of wish to die or self-harm; severe or persistent distress, defined as inability to eat or drink for >48 h, inability to sleep for >2 consecutive nights, or complete social withdrawal; and acute confusion, defined as disorientation to time, place, or person.

On identification of any red flag, the ASHA calls the CHO immediately, with an expected response within two hours. If the CHO is unreachable within 30 min and the situation is acutely life-threatening, the ASHA activates the national ambulance service (112) directly. Emotional red flags without immediate safety risk are managed through CHO-coordinated psychiatric referral within 24 h. Cases requiring specialised care will be referred to higher-level facilities, including district-level psychiatric services for complex emotional conditions.

All red-flag events, referral actions, and outcomes are documented in the C-COMPASS register column. All operational definitions, the scripted self-harm probe, the referral decision algorithm, and expected response times are addressed during the mandatory full-day ASHA training through didactic instruction and role-play scenarios, and fidelity to red-flag documentation is assessed monthly through the CHO supervision checklist.

Early neonatal deaths will be identified through the same facility-based notification pathway.

#### 2.6.4 Reporting, documentation, and ASHA training

ASHAs will document the stillbirth notification date, visit dates, support provided, red flags identified, and referrals made in an additional C-COMPASS column integrated into existing ASHA registers.

CHOs will compile and report monthly summaries, including total stillbirths, number counselled, and number referred, to the Primary Health Centre (PHC). These indicators will be incorporated into routine postnatal care (PNC) reporting systems, requiring no additional data platform.

ASHA training will be conducted as a structured half-day session covering the 3-Circle Healing Model, use of scripted counselling approaches (including direct enquiry for self-harm), identification of red flags, referral pathways, and documentation procedures. Training will be delivered by CHOs across both intervention blocks prior to implementation.

### 2.7 Block selection and matching

Four of the six SHRiSTI-surveilled blocks will be purposively selected: two allocated to the intervention arm (receiving C-COMPASS from May 2026) and two to the control arm (routine postnatal care). Block pairs will be matched based on population size, ASHA density, monthly stillbirth volume from SHRiSTI surveillance, distance to district referral facilities, and absence of any co-occurring community mental health programme.

Women in the control blocks will receive routine postnatal care as currently delivered within the National health mission program, without any C-COMPASS protocol, training, or structured referral pathway. In practice, ASHA postnatal visits in this context are designed for women with live births and focus on newborn care and breastfeeding support; no standardised protocol, training, or referral pathway exists for ASHA-delivered emotional support following stillbirth. Evidence from qualitative research conducted in the same district using a social autopsy lens found that postnatal support for bereaved women is largely informal and inconsistent, with women commonly discharged from facilities within 24–48 h with minimal counselling and returning to home environments where grief is frequently met with social silence or pressure rather than structured acknowledgement (8). No scripted emotional support, self-harm enquiry, lactation suppression guidance, or structured psychological referral pathway is routinely activated following stillbirth in the control blocks. The Tele-MANAS helpline is available as national infrastructure but is not routinely provided to bereaved women in current practice. The control condition thus represents the documented current standard of care in this setting, against which the incremental effect and cost of C-COMPASS will be estimated.

The remaining two blocks, under SHRiSTI project, will be excluded to minimise contamination, address time and resource constraints, and allow their use as potential future comparators.

### 2.8 Outcomes

#### 2.8.1 Primary effectiveness outcome

The primary effectiveness outcome will be the mean Edinburgh Postnatal Depression Scale (EPDS) total score (continuous, range: 0–30) at Day 60 postpartum (±2 days), measured by an independent study nurse (15). The EPDS was selected as the primary outcome on the basis that the primary target of C-COMPASS is the prevention of pathological depressive disorder following stillbirth, rather than grief resolution *per se*, a longer-term outcome beyond the scope of this first evaluation. The potential overlap between grief and depressive symptoms in the early postpartum period is acknowledged; this is addressed within the study design by the concurrent administration of the Brief Grief Questionnaire (BGQ) as a secondary outcome, enabling exploratory analysis of the relationship between grief and depression constructs in this population. The EPDS has been used in perinatal loss populations and validated in Indian perinatal settings (16, 17), and its use as the primary outcome enables direct comparison with the closest available LMIC analogue and alignment with NHM mental health referral thresholds.

Outcome assessment will be scheduled at Day 60, after the ASHA's Day 42 visit, to ensure a minimum three-day separation between the final C-COMPASS contact and outcome measurement, thereby minimising response priming and maintaining assessor independence. The EPDS captures symptoms over the preceding seven days; thus, administration at Day 60 reflects the post-intervention steady state. This timing is consistent with the original validation of the EPDS at 6–8 weeks postpartum (15).

Where Verbal Autopsy (VASA) is conducted for the same participant, EPDS, SDS, and BGQ will be administered first, preferably on a different day. If same-day administration is unavoidable, outcome measures will be completed prior to VASA to minimise recall-induced emotional bias.

#### 2.8.2 Secondary effectiveness outcomes

- EPDS ≥ 13 (probable depressive illness), included as a pre-specified binary outcome for policy-relevant interpretation. The study is not powered for confirmatory testing of this threshold; results will be reported with 95% confidence intervals as exploratory evidence (24).
- Sheehan Disability Scale (SDS) total score (0–30), assessing functional impairment across work/school, social, and family domains (18).
- Brief Grief Questionnaire (BGQ) score, assessing risk of complicated grief (19).

All secondary outcomes will be measured at Day 60 (±7 days), postpartum by an independent study nurse.

#### 2.8.3 Implementation outcome

The implementation outcome will be the 72 h ASHA visit coverage rate, defined as the proportion of eligible women in intervention blocks receiving a first ASHA visit within 72 h of hospital discharge. This will be measured using ASHA registers and CHO supervision records.

This indicator is expected to be a key determinant for policy adoption. The Supplementary Table S1 details the operational definitions, and implementation outcomes assessed for this study.

#### 2.8.4 Implementation outcome framework

Implementation outcomes will be assessed using the Proctor et al. (13) taxonomy, including acceptability, adoption, appropriateness, feasibility, fidelity, penetration, cost (incremental cost per woman counselled; see Section 2.8), and sustainability (including ASHA intention to continue beyond the study period).

### 2.9 Incremental cost analysis

An incremental cost analysis will be conducted from the health system perspective to estimate the additional cost of delivering C-COMPASS per woman counselled, compared to routine postnatal care. A full cost-effectiveness analysis will not be undertaken at this stage, as reliable effectiveness estimates and utility-based outcomes (e.g., QALYs or DALYs) are not yet available. The analysis is intended to inform programmatic decision-making by estimating the additional resources required for implementation.

Three cost components will be included.

First, ASHA time costs per case will be estimated based on self-reported time spent on each contact (72 h visit, Day 7 telephone call, Day 14 visit, and Day 42 visit), multiplied by an imputed hourly rate derived from the average monthly ASHA incentive.
Second, direct ASHA costs will include out-of-pocket expenditures such as transport and printed materials incurred during service delivery.
Third, programme-level costs will include ASHA training costs (trainer time and ASHA participation time, amortised over three years and allocated per expected annual caseload), CHO supervision time, nurse/ANM time for activities and any additional printing or documentation costs.

The primary cost metric will be the incremental cost per woman counselled (INR). Secondary cost indicators will include cost per woman screened positive for depression and cost per referral completed. Cost data will be collected using a structured costing tool administered to ASHAs and CHOs at the end of the study period. Routine care costs in control blocks will be estimated to provide comparative context. All costs will be reported in 2026 Indian Rupees (INR), and no discounting will be applied given the short study duration. Findings will be interpreted in relation to existing benchmarks for community-based mental health interventions in low- and middle-income settings to support policy relevance.

### 2.10 Implementation framework and strategy selection

The selection of implementation science frameworks for C-COMPASS was guided by the Systematic Evaluation and Selection of Implementation Science Theories, Models and Frameworks (SELECT-IT) meta-framework, which provides a structured approach for aligning theories, models, and frameworks with study purpose.

Based on this process, three complementary frameworks were selected. The Theoretical Domains Framework (TDF) will be used to identify behavioural and contextual determinants influencing ASHA delivery. The ERIC (Expert Recommendations for Implementing Change) taxonomy will be used to specify and classify implementation strategies embedded within the intervention. Implementation outcomes will be assessed using the Proctor et al. (13) taxonomy, given its suitability for early-stage evaluations where effectiveness is not yet established.

### 2.11 Implementation strategy specification

Implementation strategies embedded within C-COMPASS will be specified using the ERIC taxonomy. These include training, use of educational materials, supportive supervision, audit and feedback, and identification of local champions.

These strategies are integrated within routine health system workflows, including ASHA training, CHO-led supervision, and register-based monitoring. Their application is aligned with identified behavioural and system-level determinants and will be evaluated alongside implementation outcomes.

A detailed mapping of ERIC strategies, their operationalisation, and corresponding determinants is provided in Supplementary Table S2.

### 2.12 Sample size

Enrolment estimates are derived from SHRiSTI ASHA pregnancy surveillance data which indicates a mean of 4.6 stillbirths per block per month (range: 3.4–5.6), with a seasonal peak during July–October (5.2–5.6 per block per month) *(surveyed data from SHRiSTI project)*. This pattern is consistent with published systematic review by Lakhoo et al. on heat-associated stillbirth risk in South Asian settings in 2025. As described in Section 2.1, the SHRiSTI community-based surveillance estimate of 17.2 per 1,000 total births is used for planning, as it more accurately captures the true community-level burden than the HMIS facility-based figure.. The per-block estimate from the same surveillance data is 4.6 per month and this was used as a base for sample size. The study will employ a difference-in-differences (DiD) design across four blocks (two intervention, two control). With two blocks per arm, the expected enrolment rate is approximately 9.2 women per arm per month. Over the 8-week pre-intervention period, approximately 17 women per arm are expected, and over the 26-week post-intervention period, approximately 56 women per arm are expected, yielding a total sample of approximately 146 women.

The primary outcome is mean EPDS score (continuous). Assuming a clinically meaningful difference of 3.5 points, a pooled standard deviation of 6.5, two-sided alpha of 0.05, and 80% power, a minimum of 55 women per arm in the post-intervention period is required. The expected sample size of 56 per arm provides approximately 81% power. To account for an anticipated 10% attrition rate, the post-intervention data collection period may be extended to ensure a minimum effective sample size of 56 women per arm.

A pre-specified sensitivity analysis including early neonatal deaths (<24 h) is expected to add approximately 18 cases per arm. This estimate is based on a neonatal mortality rate of ∼22 per 1,000 live births in Haryana, with approximately 25% of deaths occurring within 24 h, applied to an estimated 277 live births per block per month. This yields approximately 1.5 early neonatal deaths per block per month, resulting in ∼18 additional cases per arm over the study period. The resulting post-intervention sample of approximately 74 per arm would provide approximately 91% power.

The secondary binary outcome (EPDS ≥ 13) is not used for power calculation. At an estimated baseline prevalence of 40%, the study would have approximately 44% power to detect a 16 percentage-point reduction. This analysis is therefore exploratory, and results will be reported with 95% confidence intervals to inform future cluster-randomised trial design.

### 2.13 Statistical analysis

The primary Difference-in-Differences (DiD) model will be specified as a linear regression with block fixed effects:

$$EPDS=\beta_{0}+\beta_{1}(Arm)+\beta_{2}(Time)+\beta_{3}(Arm\times Time)+\gamma_{k}(Block_{k})+\epsilon$$

where *Arm* (1 = intervention, 0 = control), *Time* (1 = post, 0 = pre), and the interaction term (*Arm* × *Time*) represents the DiD estimator (*β*₃). A negative *β*₃ will indicate a reduction in depressive symptoms attributable to C-COMPASS. Block fixed effects (*γ*_k_) will account for time-invariant block-level confounders, including baseline ASHA performance, population characteristics, and facility access.

The pre-specified secondary binary outcome (EPDS ≥ 13) will be analysed using a logistic DiD model with the same structure. SDS and BGQ scores will be analysed using linear DiD models with block fixed effects, while BGQ screen-positive (≥4) will be analysed using logistic DiD models. Implementation outcomes (visit completion, referral completion, and documentation completeness) will be reported as proportions with 95% confidence intervals.

Parallel trends will be assessed by comparing pre-intervention EPDS distributions across study arms and blocks, along with baseline sociodemographic characteristics (20). A maximum of one additional covariate beyond block fixed effects will be included, selected *a priori* (most likely gravida or previous stillbirth) (21). A pre-specified sensitivity analysis will replicate the primary model in an expanded population including early neonatal deaths, incorporating a perinatal loss type indicator and its interaction with the intervention effect. All analyses will be conducted using Stata version 16 or R version 4.4+.

### 2.14 Qualitative data analysis

Qualitative data from ASHA and CHO key informant interviews, ASHA feasibility matrix open responses, and Module I responses from enrolled women will be analysed using the Framework Method by (31) and (32).

Framework analysis is selected as it is well suited to applied health systems research, enables systematic comparison across study arms and implementation roles, and supports the integration of deductive and inductive coding approaches. An *a priori* coding framework will be developed using the 14 domains of the Theoretical Domains Framework (TDF) as primary codes and the Proctor implementation outcome domains as secondary codes, enabling linkage between determinants and implementation outcomes. Analysis will follow five stages: familiarisation, development of the analytical framework, indexing, charting, and mapping and interpretation. Two independent researchers will code all data, with discrepancies resolved through consensus.

All qualitative analysis will be conducted using NVivo version 14. Reporting will follow the COREQ (Consolidated Criteria for Reporting Qualitative Research) checklist.

### 2.15 Ethics and dissemination

This study is embedded within the ongoing SHRiSTI implementation research. The SHRiSTI protocol has been approved by the Ethics Committee of the Society for Applied Studies (Approval No. ERC: SAS/ERC/ICMR Stillbirths Study/2024; approval date: January 24, 2024). Written informed consent will be obtained from all participating women and healthcare providers following explanation of the study procedures. Ethical approval includes permission to identify and contact potential participants using routine health system records, with all consent and data collection procedures conducted by independent research staff rather than frontline health workers. Participation will be voluntary, and refusal to participate will not affect access to routine health services. Participants may withdraw at any time or decline audio recording.

Women in control blocks receive the same outcome assessment as intervention block women. Routine care in control blocks represents the current standard of care. Any participant requiring clinical attention at the Day 60 assessment is referred by the study nurse regardless of block assignment. Any non-zero response on EPDS item 10 triggers immediate escalation: the study nurse contacts the supervising research team physician, activates referral to CHO and district psychiatrist. This protocol applies in all four blocks. Tele-MANAS helpline cards are provided to all enrolled women. Only de-identified data will be used for analysis for maintain the confidentiality and the data will be accessed only by Principle investigator (PI) and CO-PI of study and necessary approval will be required for access.

Findings will be disseminated through peer-reviewed publication, presentation to the Haryana State Health Mission and national level dissemination and open-access data deposit in a publicly accessible repository upon study completion, in accordance with open science principles.

This protocol is reported in accordance with the Standard Protocol Items: Recommendations for Interventional Trials (SPIRIT) guidelines (Supplemntary Annexure 1). This manuscript additionally follows the Standards for Reporting Implementation Studies (StaRI) checklist, given the concurrent implementation evaluation embedded within the Hybrid Type I design; the completed StaRI checklist is provided as Supplemntary Annexure 2.

## 3 Discussion

C-COMPASS represents, to our knowledge, the first structured, community-health-worker-delivered psychosocial care protocol specifically designed for women following stillbirth in India to be subjected to a formal prospective evaluation using an implementation science framework. The significance of this goes beyond filling a programmatic gap. India contributes approximately one-quarter of the global stillbirth burden, an estimated 460,000 losses annually, yet no component of the NHM postnatal care framework addresses the psychological needs of women after perinatal loss (1, 2). This is not a resource problem. The SHRiSTI surveillance finding that Palwal's true community-level stillbirth rate of 17.2 per 1,000 total births is approximately 1.8-fold higher than the HMIS facility-based figure of 9.4 per 1,000 live births is itself a significant public health finding, underscoring the inadequacy of facility-based ascertainment for perinatal mortality in India and the urgency of a community-level response that reaches women who never appear in facility registers and ASHAs are already present, already visiting, and already trusted. The gap is structural: the absence of a protocol, a training module, and a referral pathway that would convert routine postnatal contact into a meaningful care response. C-COMPASS is designed to fill exactly this gap without requiring any new cadre of worker, any new data system, or any new government infrastructure. Critically, the study does not merely describe the intervention, it evaluates it in a real-world district health system, using empirical SHRiSTI surveillance data for sample size estimation, real block-level implementation conditions, and independent outcome assessment. The co-design process, grounded in five iterative consultation cycles within SHRiSTI involving bereaved women, ASHAs, CHOs, district psychiatrists, and national programme leads, ensures that C-COMPASS is not a protocol adapted from a high-income context but one built from and for the communities it serves. Beyond effectiveness, this study will generate the first empirical data on whether India's ASHA workforce can achieve systematic fidelity to a structured counselling protocol at scale, evidence that no amount of efficacy data from controlled trial settings can provide, and that is the decisive input for any government decision on national programme integration.

There are strong theoretical and empirical grounds to expect that C-COMPASS will reduce postpartum depressive symptoms at 60 days. The intervention targets the acute vulnerability window: the 72 h following hospital discharge-a period identified across multiple studies as critical for the trajectory of grief and depression after perinatal loss (6, 25, 26). Early structured emotional acknowledgement, in the absence of blame or silence, interrupts the social isolation that amplifies depressive symptomatology in the weeks that follow (8, 9). The four-contact structure mirrors the phased support model demonstrated to be effective in comparable LMIC community mental health interventions. Rahman and colleagues showed a mean EPDS reduction of 3.8 points using a community-health-worker-delivered cognitive behavioural intervention in rural Pakistan (22); Tripathy and colleagues demonstrated reductions of 3.0–4.2 points through participatory women's group programmes in Jharkhand and Orissa (23). These are the closest available analogues, and both used ASHAs or equivalent frontline workers. The effect size assumed for C-COMPASS, a 3.5-point difference in mean EPDS score, is consistent with this evidence base and is grounded in the documented EPDS distributions in Indian perinatal populations (16, 17). Moreover, the structured red-flag referral pathway, including scripted suicidal ideation enquiry at Day 7, addresses the subset of women at risk of severe outcomes, a clinically important component that no existing routine postnatal programme currently provides for bereaved women in India.

The implementation case for C-COMPASS rests on a single core argument: it is designed to be absorbed into the existing system, not added onto it. India's 1.04 million ASHAs are already mandated to conduct postnatal home visits under the Home-Based Newborn Care (HBNC) programme (10). C-COMPASS converts the visit that ASHAs already make after a stillbirth, currently an unstructured, informal, and inconsistent interaction, into a structured, documented, and evaluated care episode. The marginal resource requirement is a half-day training, a Hindi job aid, and a column in the existing ASHA register. The Tele-MANAS referral pathway (1800-891-4416) is already operational national infrastructure. The incremental cost analysis embedded in this study will quantify this argument in INR per woman counselled, the metric that government decision-makers require before considering programme adoption. The incremental cost analysis will determine whether C-COMPASS falls within the ₹200–500 per beneficiary threshold typical of scalable LMIC community health programmes. If C-COMPASS achieves the feasibility threshold of ≥70% 72 h visit coverage across both intervention blocks, it will demonstrate that ASHAs with targeted training can reliably identify and reach bereaved women in the critical early postpartum window, a finding that has direct programme planning implications at state and national level.

Bereavement care after stillbirth in high-income countries has been substantially developed over the past two decades. Guidelines from the International Stillbirth Alliance, the ACOG toolkit, and the SANDS bereavement framework provide detailed protocols for communication at the time of loss, memory-making support, and specialist psychological follow-up (5). These models are delivered by bereavement midwives, perinatal mental health specialists, and dedicated follow-up clinics, resources that do not exist and cannot be assumed in rural district hospitals in Haryana. They were not designed for LMIC delivery, and direct adaptation has consistently failed to produce implementable models for low-resource frontline settings. The closest analogue in the Indian context is the Thinking Healthy Programme, which trained community health workers to deliver a cognitive behavioural intervention for perinatal depression, but this was developed for postnatal depression after live birth, not perinatal loss, and required a 10-day training programme and structured supervision that exceeds what is feasible within routine NHM operations (22). C-COMPASS occupies a distinct and underserved position: it is the first protocol designed specifically for post-stillbirth care, delivered through the community health worker infrastructure that already exists at scale, with a training burden and documentation requirement calibrated to real ASHA capacity. The 3-Circle Healing Model at its core: Heart, Mind, Support, was developed and validated through the SHRiSTI co-design process, not translated from a Western psychotherapy framework. This distinction matters both scientifically and practically: an intervention designed for its delivery context is more likely to achieve fidelity, acceptance, and sustainability than one adapted from a structurally different system. Globally, C-COMPASS is distinct in three ways that no existing post-stillbirth protocol combines: it is delivered through a cadre of community health workers (ASHAs) who are trusted members of the same communities they serve; it provides structured grief counselling grounded in a co-designed indigenous healing framework (the 3-Circle Model) rather than an adapted Western psychotherapy protocol; and it operates within and is evaluated as part of a functioning public health system, making its findings directly transferable to government programme adoption rather than requiring a separate scale-up translation phase. Finally, it is worth noting that the concentration of enrolment in the July–October post-intervention period, which corresponds to the seasonal peak in stillbirths documented in SHRiSTI surveillance data, is expected to support timely achievement of the target sample size within the December 2026 data collection deadline, a pattern consistent with published evidence on heat-associated increases in stillbirth risk during peak temperature months in South Asian settings (27).

### 3.1 Strengths and limitation

The study has several methodological and contextual strengths. First, the use of validated, multi-domain outcome measures, EPDS for depressive symptoms, SDS for functional impairment, and BGQ for complicated grief risk, provides a comprehensive and clinically meaningful picture of recovery at 60 days, going beyond single-domain assessments used in most comparable studies. Second, the separation of outcome assessment from intervention delivery, with independent study nurses administering all scales at Day 60 after the ASHA's Day 42 contact, minimises ascertainment bias and response priming, a methodological safeguard rarely implemented in community-based LMIC trials. Third, the four-block DiD design with block-level fixed effects provides a more rigorous quasi-experimental estimate than a single-site comparison, controlling for time-invariant block-specific confounders and enabling a more robust parallel trends assessment. Fourth, the sample size is grounded in empirical SHRiSTI ASHA surveillance data from 895 ASHAs across all Palwal blocks rather than published estimates or assumptions, producing a more realistic and defensible enrolment projection. Fifth, the systematic framework selection process, guided by the SELECT-IT meta-framework, ensures that each theoretical tool, TDF for barriers and facilitators, ERIC for strategy specification, Proctor for evaluation, is chosen against explicit criteria of purpose, attributes, and project fit, rather than by convention. Finally, embedding the study within a functioning implementation research programme ensures that implementation metrics reflect genuine health system performance rather than research-assisted delivery, which is essential for generating evidence that is usable by government programme managers.

Several limitations must be acknowledged. The quasi-experimental DiD design, while appropriate given the operational constraints of embedding evaluation within a running programme, does not provide the causal certainty of a cluster-randomised trial. Block-level allocation is determined by programme implementation schedule rather than random assignment, and unmeasured time-varying confounders at block level, such as a community event, a local health programme, or seasonal migration patterns differentially affecting one block, cannot be fully excluded, even with block fixed effects. The pre-intervention period of approximately eight weeks is short, which limits formal statistical testing of the parallel trends' assumption; while the four-block design partially mitigates this, the concern cannot be eliminated. The primary sample of approximately 146 women is sufficient for the continuous EPDS primary outcome at 81% power but is underpowered for confirmatory testing of the binary EPDS ≥ 13 threshold, which should be interpreted as exploratory and directional. ASHAs in the intervention blocks are not blinded to the nature of the intervention, introducing the possibility of social desirability effects in self-reported implementation metrics, particularly visit completion and documentation completeness. The EPDS was originally validated for postnatal depression after live birth; while it has been used in stillbirth populations across multiple settings, its psychometric properties specifically in the context of perinatal grief in rural North India have not been formally validated, and cultural norms around mourning, including the social expectation that a bereaved woman will not laugh or plan for the future, may influence item-level responses in ways that are not fully separable from clinical depression. A residual risk of exclusion remains for women who experience stillbirth entirely outside facility or ASHA notification pathways, including those in the most geographically isolated, temporary migrants and women who relocate immediately after delivery. While the dual notification design and backup ANM pathway minimise this risk, penetration data collected during the study will allow estimation of the proportion of identifiable cases successfully enrolled, and any systematic differences between enrolled and non-enrolled women will be reported. Finally, the study is conducted in a single district in one state; while Palwal's epidemiological profile is broadly consistent with high-burden rural North Indian settings, immediate generalisability to other states and contexts should not be assumed.

### 3.2 Policy implications and future research

This study is designed from the outset to generate evidence that is actionable within India's government health system. The outcome measures, mean EPDS score, 72 h visit coverage rate, three-visit completion, referral completion, align directly with NHM programme monitoring indicators. The documentation pathway, a C-COMPASS column in the existing ASHA register, requires no new technology and no new reporting layer. The Tele-MANAS referral is already national infrastructure. If C-COMPASS achieves feasibility (≥70% 72 h coverage) and demonstrates a clinically meaningful reduction in mean EPDS score, the findings will be presented to the Haryana State Health Mission with a costed phased scale-up plan covering all six Palwal blocks, and subsequently to the national NHM technical advisory group for maternal mental health. The incremental cost analysis will provide the economic argument: if C-COMPASS can be delivered at a per-woman cost comparable to other NHM community interventions, the case for national integration becomes substantially stronger. The pre-specified sensitivity analysis for early neonatal deaths addresses the policy question of whether a single protocol can serve the full perinatal mortality framework population, if the answer is yes, the scope of the programme and its potential impact more than doubles without requiring protocol redesign. Future research priorities include a fully powered cluster-randomised controlled trial with the administrative block as the unit of allocation and a pre-specified intra-cluster correlation coefficient; longitudinal follow-up at 6 and 12 months to assess persistence of effects on grief and functional recovery; a formal health economic evaluation incorporating DALY metrics and multi-year programme costs; and deliberate protocol adaptation studies for early neonatal death, late miscarriage, and preterm birth with subsequent infant death, each requiring their own co-design process and piloting before evaluation. Also, systematic reviews and meta-analysis to generate evidence on effective interventions for promoting mental health in the reproductive continuum must be undertaken (28). The implementation science approaches applied in C-COMPASS, including quasi-experimental design for programme evaluation, mixed-methods feasibility assessment, and health systems integration, build on methodological frameworks previously applied to community-based child health programme implementation in comparable North Indian settings, where structured implementation research has demonstrated the value of embedding evaluations within existing health system infrastructure to generate actionable evidence for national programme scale-up (29, 30, 31).
