# Supplementary material for: C-COMPASS: protocol for a quasi-experimental hybrid type I effectiveness-implementation study of community-based compassionate care after stillbirth in India
Source: Front Health Serv. 2026 Jul 14;6:1879371. doi: 10.3389/frhs.2026.1879371 (PMC13407621; doi:10.3389/frhs.2026.1879371)
Supplement: Supplementary file 1 [file Datasheet1.pdf]

SPIRIT 2025 checklist of items to address in a randomized trial protocol

Section / Topic	No	SPIRIT 2025 checklist item description	Reported on page no.
**Administrative information**			
Title and structured summary	1a	Title stating the trial design, population, and interventions, with identification as a protocol	Page 1, Title identifies intervention, population, and protocol design
	1b	Structured summary of trial design and methods, including items from the World Health Organization Trial Registration Data Set	Page 2 & 3, Structured abstract includes background, methods, discussion, and registration
Protocol version	2	Version date and identifier	Reported on page 16
Roles and responsibilities	3a	Names, affiliations, and roles of protocol contributors	Page 1, and Page 22, Authors, affiliations, and contributions described
	3b	Name and contact information for the trial sponsor	Page 1, and Page 21-22, funding institution details
	3c	Role of trial sponsor and funders in design, conduct, analysis, and reporting of trial; including any authority over these activities	Page 22, States funder had no role in design, analysis, or reporting
	3d	Composition, roles, and responsibilities of the coordinating site, steering committee, endpoint adjudication committee, data management team, and other individuals or groups overseeing the trial, if applicable	Page 8-10, Roles of district health authorities, ASHAs, CHOs, ANMs, and study supervisors described
**Open science**			
Trial registration	4	Name of trial registry, identifying number (with URL), and date of registration. If not yet registered, name of intended registry	
Protocol and statistical analysis plan	5	Where the trial protocol and statistical analysis plan can be accessed	Page 15-16
Data sharing	6	Where and how the individual de-identified participant data (including data dictionary), statistical code, and any other materials will be accessible	Page 17, Mentions open-access data deposit after study completion
Funding and conflicts of interest	7a	Sources of funding and other support (e.g., supply of drugs)	Page 21 , 22, Funding source and grant number described
	7b	Financial and other conflicts of interest for principal investigators and steering committee members	Page 22, Lines 13–15: Conflict of interest declaration
Dissemination policy	8	Plans to communicate trial results to participants, healthcare professionals, the public, and other relevant groups (e.g., reporting in trial registry, plain language summary, publication)	Page 16-17, Dissemination through publication, presentations, and open-access repository
**Introduction**			
Background and rationale	9a	Scientific background and rationale, including summary of relevant studies (published and unpublished) examining benefits and harms for each intervention	Page 4–5, throughout: Burden of stillbirth, psychosocial impact, evidence gaps, and rationale described

	9b	Explanation for choice of comparator	Page 10 and Page 16, Routine postnatal care used as comparator/current standard of care
Objectives	10	Specific objectives related to benefits and harms	Page 6, and Page 7, Primary and secondary objectives listed
**Methods: Patient and public involvement, trial design**			
Patient and public involvement	11	Details of, or plans for, patient or public involvement in the design, conduct, and reporting of the trial	Page 7-8, and Page 17, Lines 5–10: Co-design involved bereaved women and stakeholders
Trial design	12	Description of trial design including type of trial (e.g., parallel group, crossover), allocation ratio, and framework (e.g., superiority, equivalence, non-inferiority, exploratory)	Page 5-6, Hybrid Type I quasi-experimental Difference-in-Differences design described
**Methods: Participants, interventions, and outcomes**			
Trial setting	13	Settings (e.g., community, hospital) and locations (e.g., countries, sites) where the trial will be conducted	Page 5–6, throughout: Palwal district, Haryana, India and health system setting described
Eligibility criteria	14a	Eligibility criteria for participants	Page 7-8, Inclusion and exclusion criteria stated
	14b	If applicable, eligibility criteria for sites and for individuals who will deliver the interventions (e.g., surgeons, physiotherapists)	Page 9: ASHAs, ANMs, and CHOs identified as intervention deliverers
Intervention and comparator	15a	Intervention and comparator with sufficient details to allow replication including how, when, and by whom they will be administered. If relevant, where additional materials describing the intervention and comparator (e.g., intervention manual) can be accessed	Page 8–10: Detailed description of four-contact C-COMPASS intervention and routine care comparator
	15b	Criteria for discontinuing or modifying allocated intervention/comparator for a trial participant (e.g., drug dose change in response to harms, participant request, or improving/worsening disease)	Page 7-10, Referral and emergency escalation procedures described
	15c	Strategies to improve adherence to intervention/comparator protocols, if applicable, and any procedures for monitoring adherence (e.g., drug tablet return, sessions attended)	Page 9-11, & 13 Register documentation, supervision, and monitoring procedures described
	15d	Concomitant care that is permitted or prohibited during the trial	Not Applicable
Outcomes	16	Primary and secondary outcomes, including the specific measurement variable (e.g., systolic blood pressure), analysis metric (e.g., change from baseline, final value, time to event), method of aggregation (e.g., median, proportion), and time point for each outcome	Page 11-14, EPDS, SDS, BGQ, implementation outcomes, and timing are clearly defined
Harms	17	How harms are defined and will be assessed (e.g., systematically, non-systematically)	Page 10-11, Physical/emotional red flags and referral process described

Participant timeline	18	Time schedule of enrollment, interventions (including any run-ins and washouts), assessments, and visits for participants. A schematic diagram is highly recommended (see Figure)	Page 7-10 and Table 1 on Page 9: Timeline of visits and assessments detailed
Sample size	19	How sample size was determined, including all assumptions supporting the sample size calculation	Page 14-15: Detailed sample size assumptions and power calculations
Recruitment	20	Strategies for achieving adequate participant enrollment to reach target sample size	Page 8-10 and Page 14-15: Surveillance-based recruitment and expected enrolment described
**Methods: Assignment of interventions**			
Randomization:			
Sequence generation	21a	Who will generate the random allocation sequence and the method used	Not applicable – quasi-experimental design
	21b	Type of randomization (simple or restricted) and details of any factors for stratification. To reduce predictability of a random sequence, other details of any planned restriction (e.g., blocking) should be provided in a separate document that is unavailable to those who enroll participants or assign interventions	Not applicable – quasi-experimental design
Allocation concealment mechanism	22	Mechanism used to implement the random allocation sequence (e.g., central computer/telephone; sequentially numbered, opaque, sealed containers), describing any steps to conceal the sequence until interventions are assigned	Not applicable
Implementation	23	Whether the personnel who will enroll and those who will assign participants to the interventions will have access to the random allocation sequence	Not applicable
Blinding	24a	Who will be blinded after assignment to interventions (e.g., participants, care providers, outcome assessors, data analysts)	Page 9-12, and Page 17,
	24b	If blinded, how blinding will be achieved and description of the similarity of interventions	Page 11-14, Outcome assessment separated from intervention delivery
	24c	If blinded, circumstances under which unblinding is permissible, and procedure for revealing a participant's allocated intervention during the trial	Not applicable
**Methods: Data collection, management, and analysis**			
Data collection methods	25a	Plans for assessment and collection of trial data, including any related processes to promote data quality (e.g., duplicate measurements, training of assessors) and a description of trial instruments (e.g., questionnaires, laboratory tests) along with their reliability and validity, if known. Reference to where data collection forms can be accessed, if not in the protocol	Page 7-10, Data collection tools, scales, and assessor procedures described
	25b	Plans to promote participant retention and complete follow-up, including list of any outcome data to be collected for participants who discontinue or deviate from intervention protocols	Page 8 Follow-up schedule

Data management	26	Plans for data entry, coding, security, and storage, including any related processes to promote data quality (e.g., double data entry; range checks for data values). Reference to where details of data management procedures can be accessed, if not in the protocol	Not stated
Statistical methods	27a	Statistical methods used to compare groups for primary and secondary outcomes, including harms	Page 15-16: Statistical analysis described
	27b	Definition of who will be included in each analysis (e.g., all randomized participants), and in which group	Page 7-8, and Page 15-16
	27c	How missing data will be handled in the analysis	Not explicitly stated
	27d	Methods for any additional analyses (e.g., subgroup and sensitivity analyses)	Page 7-8 and Page 15-16: Sensitivity analysis described
**Methods: Monitoring**			
Data monitoring committee	28a	Composition of data monitoring committee (DMC); summary of its role and reporting structure; statement of whether it is independent from the sponsor and funder; conflicts of interest and reference to where further details about its charter can be found, if not in the protocol. Alternatively, an explanation of why a DMC is not needed	Not stated
	28b	Explanation of any interim analyses and stopping guidelines, including who will have access to these interim results and make the final decision to terminate the trial	Not stated
Trial monitoring	29	Frequency and procedures for monitoring trial conduct. If there is no monitoring, give explanation	Page 10-11 and Page 16: CHO supervision and monitoring procedures described
**Ethics**			
Research ethics approval	30	Plans for seeking research ethics committee/institutional review board approval	Page 16-17, Ethics approval details provided
Protocol amendments	31	Plans for communicating important protocol modifications to relevant parties	Not stated
Consent or assent	32a	Who will obtain informed consent or assent from potential trial participants or authorized proxies, and how	Page 7, Written informed consent procedures described
	32b	Additional consent provisions for collection and use of participant data and biological specimens in ancillary studies, if applicable	Not applicable
Confidentiality	33	How personal information about potential and enrolled participants will be collected, shared, and maintained in order to protect confidentiality before, during, and after the trial	Page 16
Ancillary and post-trial care	34	Provisions, if any, for ancillary and post-trial care, and for compensation to those who suffer harm from trial participation	Page 8-10, Referral and emergency psychiatric support procedures described

This protocol is a quasi-experimental Hybrid Type I effectiveness–implementation study and not a randomized controlled trial; therefore, SPIRIT items relating specifically to randomization and allocation concealment are marked “Not applicable.”
