# Supplementary material for: C-COMPASS: protocol for a quasi-experimental hybrid type I effectiveness-implementation study of community-based compassionate care after stillbirth in India
Source: Front Health Serv. 2026 Jul 14;6:1879371. doi: 10.3389/frhs.2026.1879371 (PMC13407621; doi:10.3389/frhs.2026.1879371)
Supplement: Supplementary file 2 [file Datasheet2.pdf]

Standards for Reporting Implementation Studies: the StaRI checklist for completion

Checklist item		Reported on page #	Implementation Strategy	Reported on page #	Intervention
		Page 14	"Implementation strategy" refers to how the intervention was implemented	Page 12	"Intervention" refers to the healthcare or public health intervention that is being implemented.
**Title and abstract**					
Title	**1**	1	Identification as an implementation study, and description of the methodology in the title and/or keywords		
Abstract	**2**	2	Identification as an implementation study, including a description of the implementation strategy to be tested, the evidence-based intervention being implemented, and defining the key implementation and health outcomes.		
**Introduction**					
Introduction	**3**	4-5	Description of the problem, challenge or deficiency in healthcare or public health that the intervention being implemented aims to address.		
Rationale	**4**	4-5	The scientific background and rationale for the implementation strategy (including any underpinning theory/framework/model, how it is expected to achieve its effects and any pilot work).	4-5	The scientific background and rationale for the intervention being implemented (including evidence about its effectiveness and how it is expected to achieve its effects).
Aims and objectives	**5**	6-7	The aims of the study, differentiating between implementation objectives and any intervention objectives.		
**Methods: description**					
Design	**6**	5-6	The design and key features of the evaluation, (cross referencing to any appropriate methodology reporting standards) and any changes to study protocol, with reasons		
Context	**7**	5-6	The context in which the intervention was implemented. (Consider social, economic, policy, healthcare, organisational barriers and facilitators that might influence implementation elsewhere).		

Targeted ‘sites’	**8**	6	The characteristics of the targeted ‘site(s)’ (e.g locations/personnel/resources etc.) for implementation and any eligibility criteria.	6	The population targeted by the intervention and any eligibility criteria.
Description	**9**	8-10 & 13	A description of the implementation strategy	8-10 &13	A description of the intervention
Sub-groups	**10**	16	Any sub-groups recruited for additional research tasks, and/or nested studies are described		
**Methods: evaluation**					
Outcomes	**11**	11	Defined pre-specified primary and other outcome(s) of the implementation strategy, and how they were assessed. Document any pre-determined targets	11	Defined pre-specified primary and other outcome(s) of the intervention (if assessed), and how they were assessed. Document any pre-determined targets
Process evaluation	**12**	11-12	Process evaluation objectives and outcomes related to the mechanism by which the strategy is expected to work		
Economic evaluation	**13**	12-13	Methods for resource use, costs, economic outcomes and analysis for the implementation strategy	12-13	Methods for resource use, costs, economic outcomes and analysis for the intervention
Sample size	**14**	14	Rationale for sample sizes (including sample size calculations, budgetary constraints, practical considerations, data saturation, as appropriate)		
Analysis	**15**	7-8 (Incremental cost analysis), 12-13 (Sensitivity analysis) & 15-16 (Statistical & Qualitative analysis)	Methods of analysis (with reasons for that choice)		
Sub-group analyses	**16**	16-17	Any a priori sub-group analyses (e.g. between different sites in a multicentre study, different clinical or demographic populations), and sub-groups recruited to specific nested research tasks		

**Results**					
Characteristics	**17**	7-8	Proportion recruited and characteristics of the recipient population for the implementation strategy	7-8	Proportion recruited and characteristics (if appropriate) of the recipient population for the intervention
Outcomes	**18**	11-12	Primary and other outcome(s) of the implementation strategy	11-12	Primary and other outcome(s) of the Intervention (if assessed)
Process outcomes	**19**	13-14 & 17	Process data related to the implementation strategy mapped to the mechanism by which the strategy is expected to work		
Economic evaluation	**20**	7-8	Resource use, costs, economic outcomes and analysis for the implementation strategy	7-8	Resource use, costs, economic outcomes and analysis for the intervention
Sub-group analyses	**21**	17	Representativeness and outcomes of subgroups including those recruited to specific research tasks		
Fidelity/ adaptation	**22**	11, 17 & 19	Fidelity to implementation strategy as planned and adaptation to suit context and preferences	11,17 & 19	Fidelity to delivering the core components of intervention (where measured)
Contextual changes	**23**	20-22	Contextual changes (if any) which may have affected outcomes		
Harms	**24**	20-22	All important harms or unintended effects in each group		
**Discussion**					
Structured discussion	**25**	19-21	Summary of findings, strengths and limitations, comparisons with other studies, conclusions and implications		
Implications	**26**	21	Discussion of policy, practice and/or research implications of the implementation strategy (specifically including scalability)	21	Discussion of policy, practice and/or research implications of the intervention (specifically including sustainability)
**General**					
Statements	**27**	21-22	Include statement(s) on regulatory approvals (including, as appropriate, ethical approval, confidential use of routine data, governance approval), trial/study registration (availability of protocol), funding and conflicts of interest		
